# Fecal microbiota composition concerning body mass index and early-life factors in Mexican preschool-aged children: a cross-sectional study

**DOI:** 10.7717/peerj.21253

**Published:** 2026-06-03

**Authors:** Dolores Hernández-Rodríguez, Astrid Contreras, Guiomar Melgar Lalanne, Silvia Socorro Lara Arellano, José Enrique Meza, Cuauhtémoc Licona Cassani, Rubi Viveros

**Affiliations:** 1 Instituto de Ciencias Básicas, Universidad Veracruzana, Xalapa, Veracruz, Mexico; 2 Centro de Investigaciones Biomédicas, Universidad Veracruzana, Xalapa, Veracruz, Mexico; 3 Facultad de Enfermería, Universidad Veracruzana, Xalapa, Veracruz, Mexico; 4 Departamento de Ingeniería Celular y Biocatálisis, Instituto de Biotecnología, Universidad Nacional Autónoma de México, Cuernavaca, Morelos, Mexico

**Keywords:** Gut microbiota, Body mass index, Clinical data, Childhood, Nutritional status

## Abstract

**Background:**

The gut microbiota maturation extends beyond the first two years of life. Early perinatal and postnatal factors may influence the child’s nutritional status, which, in turn, can affect the composition of their gut microbiota.

**Methods:**

This cross-sectional study characterized the gut microbiota of 84 preschool children (ages 3 to 5) from semi-urban and urban areas in Veracruz, Mexico. Fecal samples were analyzed using 16S rRNA gene sequencing. Perinatal and early-life exposures were retrieved retrospectively using a validated, interviewer-administered questionnaire, while current anthropometric measurements and dietary patterns were evaluated. Children were classified by Body Mass Index (BMI) as underweight (UW), normal weight (NW), or overweight/obese (OW).

**Results:**

Clinical factors such as prematurity and early feeding showed weak associations with BMI. The dietary patterns exhibited general homogeneity, showing a Western diet with no significant changes in relation to BMI. The predominant bacterial phyla were Bacillota and Bacteroidota across all BMI groups. Alpha diversity showed significant differences according to BMI categories, while no significant differences in beta diversity, or the relative abundance of major phyla, were observed. At the family level, Prevotellaceae was more abundant in underweight children, while Lachnospiraceae was more prevalent in those with higher BMI. However, these trends were not statistically significant. The findings suggest that, within this narrow age range and shared environment, gut microbiota composition is broadly similar regardless of BMI and dietary patterns, highlighting the complexity of microbiota–host interactions in early childhood. Further studies integrating functional analyses are needed to clarify the causal links between gut microbiota and a child’s weight status.

## Introduction

The term gut microbiota (GM) refers to the community of microorganisms present in the gastrointestinal tract. The role that these microorganisms play in the health or disease of their hosts is determined by their composition (Afzaal et al., 2022). When the composition of the microbiota becomes unbalanced (meaning that pathogenic and opportunistic bacteria increase regarding beneficial ones), a condition known as dysbiosis arises. This imbalance can disrupt physiological functions and contribute to the development of various childhood diseases, including type-1-diabetes, allergies, necrotizing enterocolitis, obesity (Jielong et al., 2022), and attention deficit hyperactivity disorder (You et al., 2024). Therefore, the study of the taxonomic composition of gut microbiota at childhood is crucial for understanding its role in maintaining host health.

The gut microbiota development begins during intrauterine life *via* the placenta and amniotic fluid (Yang et al., 2021); however, evidence is still inconclusive. Following birth, a wide range of maternal and environmental factors, such as maternal nutritional status, type of delivery, antibiotic exposure, and breastfeeding practices, are pivotal in shaping infant microbiota (Pantazi et al., 2023). In this context, the first 1,000 days of life have received considerable attention from researchers due to their influence on the early colonization and maturation of the intestinal ecosystem (Bäckhed et al., 2015; Bokulich et al., 2016; Indrio & Marchese, 2024; Murphy et al., 2017).

Consequently, gut microbiota composition has been extensively studied in children under two years of age; however, studies in preschool-aged children are still scarce (Derrien, Alvarez & De Vos, 2019). This is crucial since gut microbiota maturation can extend up to six years in humans (Bankole & Li, 2025). During this time, gut microbiota is still susceptible to environmental factors, particularly family dietary habits, which are closely linked to the child’s nutritional status (Iddrisu et al., 2021), which is commonly evaluated based on dietary patterns and child growth standards by guidelines established by the World Health Organization (WHO) (World Health Organization, 2025).

In preschool-aged children, especially in developing countries, the influence of perinatal and postnatal factors on nutritional status and gut microbiota composition has not been widely studied. Malnutrition, encompassing both undernutrition and overweight/obesity, continues to pose a major public health challenge in low- and middle-income nations (Viana, De Araújo-Moura & De Moraes, 2025). The rate of childhood malnutrition is increasing both in Mexico and worldwide (Shamah-Levy et al., 2024; World Health Organization, 2022), emphasizing the need to implement preventive strategies focused on promoting a healthy gut microbiota from early life (Szajewska, 2025). Therefore, it is essential to understand the establishment of healthy gut microbiota beyond the first 1,000 days of life and examine how perinatal and postnatal early life factors contribute to the child’s nutritional status and thus, the gut microbiota composition.

This work characterized the gut microbiota composition of preschool-age children with overweight/obese, underweight, and normal-weight using 16S rRNA gene-targeted sequencing, considering clinical records, comprising perinatal and postnatal data, collected from a parent/caregiver questionnaire. We explore whether early-life exposures are associated with the children’s current body mass index (BMI) and, in turn, between BMI and fecal microbial composition. To reduce variation unrelated to weight status, all participants were from the same geographic area.

## Materials & Methods

### Study design and setting

We conducted a cross-sectional study and employed a census-based sampling approach. Preschoolers aged 3 to 5 years, recruited from semi-urban kindergartens in Veracruz, Mexico, between June 2022 and April 2023. The parents or guardians of children in the kindergartens of the study region were invited through an in-person information meeting. All children enrolled in the participating schools during the study period were assessed for eligibility and provided written informed consent, and child assent were enrolled and no *a priori* sample size calculation was performed; consequently, the sample size was determined by the total number of eligible participants available in the study setting. Eligibility criteria included children who had not consumed probiotics within the previous month or received antibiotic treatment. In total, 110 parents or guardians consented, and 84 of them provided a stool sample with valid 16S rRNA gene sequencing and were included in microbiota analyses. This work was approved by the research committee of the Institute of Biological Research and the ethics committee of the Institute of Psychological Research from Universidad Veracruzana, with approval numbers 21-06 and 202116, respectively.

### Sample collection

Parents of the participants were instructed, both verbal and written, to collect a stool sample at home and were provided with a stool collection kit. The kit included printed instructions, latex gloves, a wooden spatula, wax paper, ID tags, and a sterile container. Fresh fecal samples were collected by the parents and kept refrigerated (4 °C) at home following explicit instructions to place the container in a sealed plastic bag and keep it separate from food items. Samples were delivered to a laboratory within a maximum of 12 h of collection and transported in coolers with ice packs. Upon arrival at the laboratory, one g aliquots were prepared and stored at −70 °C until further analysis.

### Clinical data

Clinical data on perinatal and postnatal factors experienced by children were collected using a clinical history questionnaire developed by the research team, based on existing scientific evidence, which was administered face-to-face by health professionals from the team to parents. Clinical history was subjected to a preliminary evaluation by a series of experts, including specialists in pediatrics, epidemiology, nutrition, gastroenterology, medicine, psychology, and lactation. Despite their validation, the findings have not yet been formally published; they were considered adequate for describing the clinical factors evaluated in this study. The clinical data included prematurity defined as gestational aged at birth <37 weeks, type of birth (vaginal delivery or cesarean section), feeding in the first six months of life (exclusive breastfeeding, mixed breastfeeding, formula feeding), birth weight (very low <1.5 kg, low <2.5 kg, normal 2.5–4 kg, and high >4 kg), skin to skin contact (immediate direct contact <1 h postpartum or delayed/not performed), hospitalization at birth (stay of the newborn in the hospital after delivery exceeding the usual length of time spent together before discharge) and incubator required (indicated in cases of prematurity, low birth weight, or other clinical indications within the initial 72 h of life).

### Body Mass Index scores

All participants underwent anthropometric evaluation following the guidelines established by WHO (World Health Organization, 2025). Briefly, height was measured with a SECA 213 stadiometer and weight with a SECA 876 scale, both previously calibrated, without shoes and wearing light clothing, taking two measurements for average. According to BMI (body mass index), the children were divided into three groups: underweight (zBMI score of < −1), normal weight (zBMI score of −1 to +1), and overweight (zBMI score of >+1 to <+3). The distribution across BMI categories reflects the natural prevalence of nutritional status in the school population and was not the result of selective recruitment or sampling bias.

### Dietary patterns

Dietary intake was assessed using a validated food frequency consumption questionnaire (FFCQ) that included 81 food items representative of the most consumed foods by the Mexican population (Gaona-Pineda et al., 2018). All data were analyzed by the DIAL software (version 3.15), with adjusted nutrient values according to the Mexican food composition table and with the recommendation of children’s intake according to their age range.

### Stool DNA extraction and 16S rRNA gene amplicon sequencing

DNA extraction was performed using the Quick-DNA™ Fungal/Bacterial kit (Zymo Research, Irvine, CA, USA), according to the manufacturer’s protocol, including cell lysis, silica-column DNA purification, washing, and final elution. The purity of the DNA was determined spectrophotometrically using a Nanodrop™ (Thermo Fisher Scientific, Waltham, MA, USA). The integrity of each gDNA sample was checked using 0.7% agarose gel (p/v) electrophoresis.

Bacterial V3-V4 hypervariable regions of the 16S rRNA gene were amplified utilizing the forward primer 341F (5′-CCTACGGGDGGCWGCAG-3′) and reverse primer 805R (5′-GACTACHVGGGTATCTAATCC-3′). Each PCR reaction was carried using high-fidelity DNA polymerase, in a final volume of 25 µl, using 10 ng/µl DNA from each sample, to verify DNA quality and amplifiability.

Finally, genomic DNA samples were to send to Core Lab Genomics, Tecnológico de Monterrey, Monterrey, Nuevo León, México). The pooled amplicons and library preparation were conducted following the guidelines provided in the MiSeq System manual (Illumina, San Diego, CA, USA) and sequencing was conducted on a V3 MiSeq 622-cycle flow cell, generating 2 × 300 bp paired-end reads. A single sequencing library was prepared from each DNA sample, and no technical replicates were included. All libraries were sequenced in the same run to minimize technical variation.

### 16S rRNA gene analysis

A total of 84 samples were successfully sequenced. Single-end reads were imported to a QZA artifact compressed file and analyzed using the bioinformatic program Quantitative Insights into Microbial Ecology 2 (QIIME2) version qiime2-2020.2 (Estaki et al., 2020). Sequence quality, control trimming, and chimera removal were performed using DADA2 v1.16.23 with resultant amplicon sequence variant (ASV) clustering at 99% similarity (Callahan et al., 2016). Reads with a mean quality below this cutoff were removed. Terminal low-quality bases at the 5′ and 3′ ends were trimmed when their per-base Q-score was <Q30. Adapter and primer contamination was removed by excising known sequence motifs from the raw reads. Forward reads were trimmed by 17 bp and truncated at 240 bp, while reverse reads were trimmed by 21 bp and truncated at 200 bp based on quality score inspection. Reads containing ambiguous bases or with more than two expected errors were discarded.

Taxonomic assignment of sequences was performed with the Naïve Bayes feature classifier q2 classify-sklearn using SILVA version 132 database as a reference, with a confidence threshold of 99% (Quast et al., 2013). Rarefaction curves for each sample were calculated with QIIME 2. The R package Conquer (https://wdl2459.github.io/ConQuR/ConQuR.Vignette.html) was used to remove batch effects from the microbiota data using conditional quantile regression. Microbial counts were normalized to correct differences in sequencing depth and to ensure that comparisons are meaningful. Relative abundances were transformed using the centered log-ratio (CLR) prior to statistical analysis; therefore, the values shown correspond to centered log-ratios rather than direct proportions; abundance values as percentages and ASV values were also obtained. The feature table obtained was filtered, and diversity was analyzed using the QIIME2 phylogenetic metrics pipeline. Given that three different groups were studied, alpha diversity was calculated for each one to enable subsequent comparisons, using the Shannon index, as it accounts for both species richness and relative abundance, expressed as a quantitative value. The Pielou index was also applied to measure and compare the evenness of the abundance distribution of the species present across the study groups; comparisons between groups were made using the Mann–Whitney test. Beta diversity analyses were based on multivariate, distance-based approaches that assess differences in global community structure rather than univariate comparisons of individual taxa; these methods are less dependent on balanced group sizes and are widely used in observational microbiome studies with naturally occurring group distributions (Anderson, 2001; Savva & Ponsero, 2025). For visualization of beta diversity among groups, unweighted and weighted UniFrac distances were calculated and plotted using principal coordinate analysis (PCoA) with the R packages Phyloseq and ggplot. The Bray–Curtis distance metric and ANOSIM (999 permutations) were used, and the analysis of differential abundance at the taxonomic family level was performed with DESeq2 v1.40.2 as a statistical model, with batch correction (Clarke, 1993).

### Statistical analysis

Complementary statistical analyses to group comparisons were performed using R statistical package and PAST version 4.07 (Hammer et al., 2001). Microbial relative abundance was evaluated with the Mann–Whitney U-test. Differences in ASVs enrichment between groups were tested using the DESeq2 algorithm that corrects for varying sequencing to identify differences between different groups of children with different BMIs by adjusting for variability in the data and correcting for differences in library size, allowing for adjusted *p*-values to be obtained (Love, Huber & Anders, 2014). Abundance data at the phyla level were transformed to logarithms and analyzed with ANOVA and Kruskal-Wallis post hoc tests to observe differences between groups with different BMI, however, reduced power for comparisons involving the underweight group is acknowledged due to its small sample size. Patterns between clinical data and current BMI were explored with Principal Component Analysis (PCA) using the PAST software (v. 4.07).

## Results

### Clinical data

Perinatal and postnatal clinical factors obtained from the medical records of the participants in the study show that some of these characteristics (type of childbirth; skin-to-skin contact, hospitalization at birth, and incubator required) did not show a clear trend that allows establishing a relationship between the factors measured and BMI, even so, we were able to observe that only 20% of normal-weight children had premature gestation, of the 80% who were born full term, 84.9% had optimal birth weights (2.5–4 kg), as detailed in Table 1.

**Table 1:** Perinatal and postnatal clinical characteristics and their relationship with BMI.

	**Underweight children** **(*n* = 5)**			**Normal weight** **children** **(*n* = 53)**			**Overweight** **children** **(*n* = 26)**		
	**Female (*N* = 3)**	**Male** **(*N* = 2)**	**Total (%)**	**Female** **(*N* = 23)**	**Male** **(*N* = 30)**	**Total (%)**	**Female** **(*N* = 10)**	**Male** **(*N* = 16)**	**Total (%)**
**Premature**	2	1	60	4	7	20	5	6	42.3
**Vaginal delivery**	2	0	40	11	18	54.7	5	3	30.7
Exclusive breastfeeding^*^	1	2	60	8	16	45.2	3	5	30.8
Mixed breastfeeding^*^	2	0	40	12	13	47.2	5	7	46.1
Formula feeding^*^	0	0	0	3	1	7.6	2	4	23
**Birth weight (kg) < 1.5**	0	1	20	0	1	1.9	0	1	3.8
**<2.5**	0	0	0	2	3	9.4	1	1	7.7
**2.5–4**	3	1	80	20	25	84.9	9	13	84.6
**>4**	0	0	0	1	1	3.8	0	1	7.7
**Skin to skin contact**	1	2	60	12	19	58.3	5	8	50
**Hospitalization at birth**	1	1	40	2	4	11.3	1	3	15.4
**Incubator required**	1	1	40	3	4	13.2	0	2	7.7

**Notes.**

* First 6 months.

**Figure 1:**
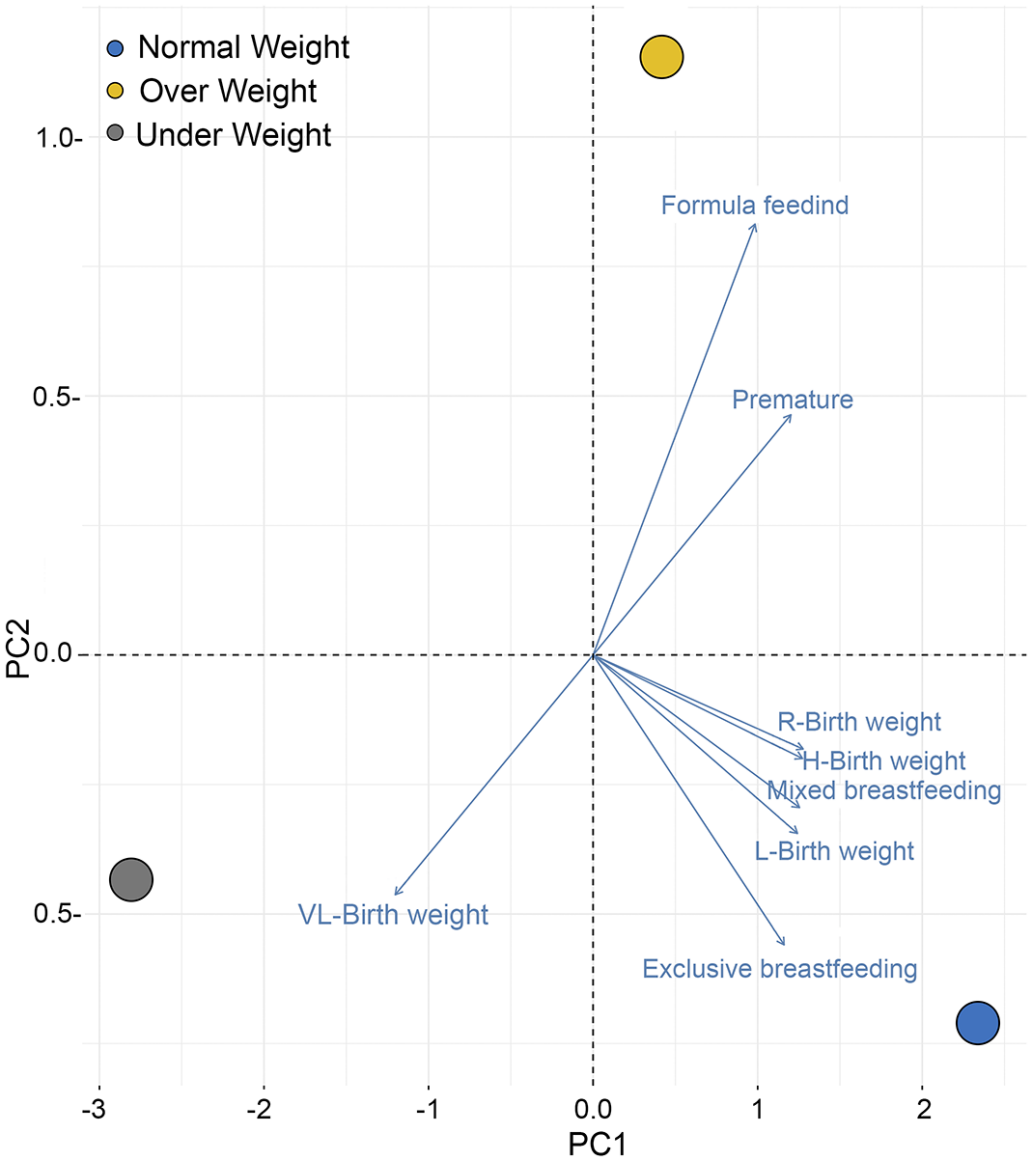
PCA of clinical data from de birth to 6 months age in relation to current BMI groups in preschool children. The clustered between normal weight and exclusive breastfeeding is highlighted. Birth weight (Kg) are presented as follows (VL-Birth weight ≤1.5, L-Birth weight ≤2.5, R-Birth weight = 2.5–4, H-Birth weight ≥4). Created with ggplot 2 in R.

PCA revealed a normal weight child (NW) were positioned closer to exclusive breastfeeding, and to a lesser extent between normal weight and mixed feeding. Also, formula feeding clustered neared to children in the overweight category. There does not seem to be such a marked influence regarding birth weight, since all birth weight categories were clustered with children with NW, except those with very low birth weight (<1.5 kg), who tended to be underweight in preschool. Contrary to what might be assumed, this low weight did not have the same trend as prematurity, which was grouped with overweight children (Fig. 1).

### Body mass index scores

Of the 84 children between 3 and 5 years old who completed the anthropometric measurements and delivered the biological samples, 48 (57.14%) were male and 36 (42.86%) were female. According to the body condition data obtained (weight/height ratio), did not significant differences between sexes (*p* = 0.709) were found in normal-weight children. The median (interquartile range) BMI was 15.33 (13.81–16.97) with a mean of 15.06 ± 0.22 in the normal-weight children (NW), 17.99 (16.74–21.29) with a mean of 16.13 ± 1.67 in the overweight (OW), and 13.16 (12.33–13.82) with a mean of 14.9 ± 0.67 for underweight (UW) (see Table S1).

### Dietary patterns

**Figure 2:**
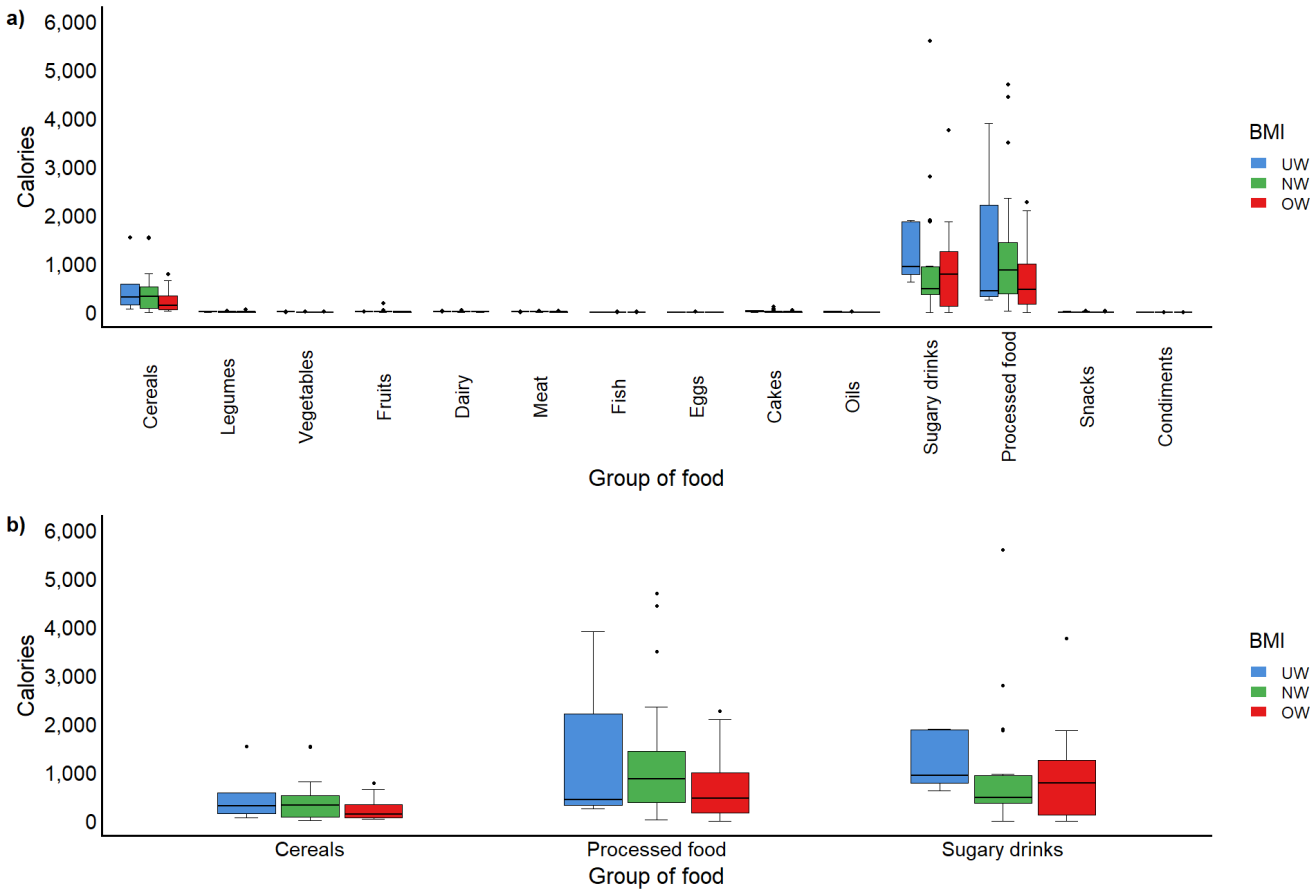
Food group consumption according to BMI categories. (A) Overall consumption of all evaluated food groups. (B) Consumption of most frequently reported food groups. Created with R studio Software (version 4.5.3; R Core Team, 2026) in Rstudio version 2026.1.2. Posit, PBC).

Dietary analysis was available for 79 of the 84 participants due to incomplete records in five cases. The energy intake of the subjects was measured by food groups, and differences between BMI categories were assessed using the Kruskal–Wallis test. Dietary analysis revealed that energy intake from diverse food groups consumed did not exhibit significant differences across BMI categories (*p* > 0.05) (see Table S2). This finding suggests that all participants showed similar dietary patterns independent of their BMI. However, despite there being no significant differences between the BMI groups, all children exhibited low consumption of fruits and vegetables and high consumption of ultra-processed foods, refined grains, and sugary drinks, characteristic patterns of Western diets (Fig. 2).

### Alpha diversity

**Figure 3:**
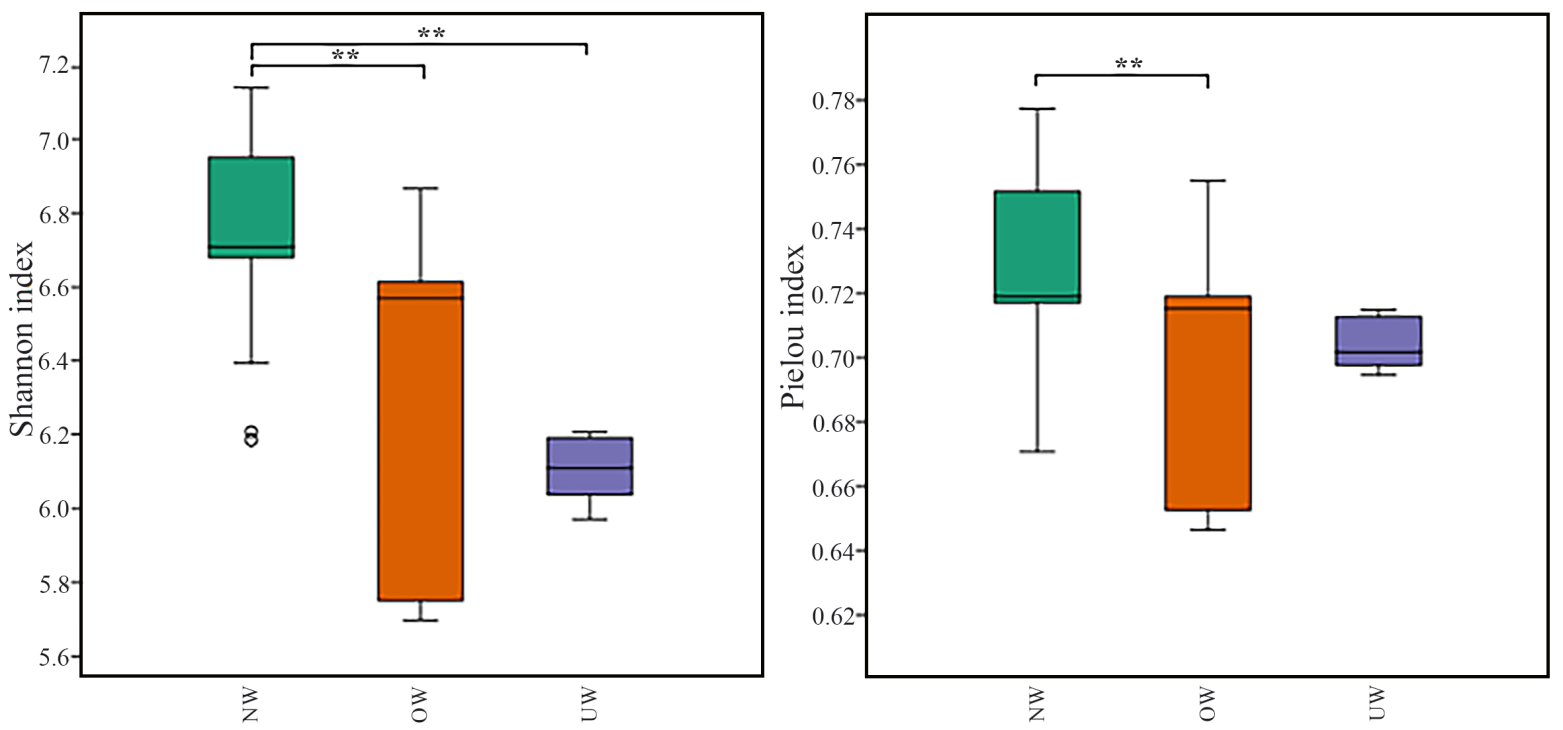
Alpha diversity according to BMI categories. Alpha diversity was assessed using the Shannon diversity index and Pielou’s evenness index. Boxplots represent the distribution of alpha diversity across BMI categories: normal weight (NW), overweight (OW), and underweight (UW). Statistically significant differences between groups are indicated (**p < 0.01). Created with QIIME 2, plugin q2-diversity (qiime diversity alpha-group-significance).

The number of ASVs was obtained per sample, ranging from 0 to 61,599, generally being higher for Bacillota, regardless of BMI (Table S3). Alpha diversity is often considered a method to describe richness, evenness, or diversity within a sample, in this case, microbial diversity. Figure 3 shows significant differences between species richness and phylogenetic diversity. The Shannon index showed greater species richness, diversity, and evenness in the NW group, with significant differences compared to the OW and UW groups (*p* < 0.001). The Pielou index indicated that all three groups reflected bacterial communities with a relatively balanced species distribution, especially in children of the NW group, who showed significant differences regarding the OW group (*p* < 0.001).

### Relative abundance of phylum in stool samples by BMI

**Figure 4:**
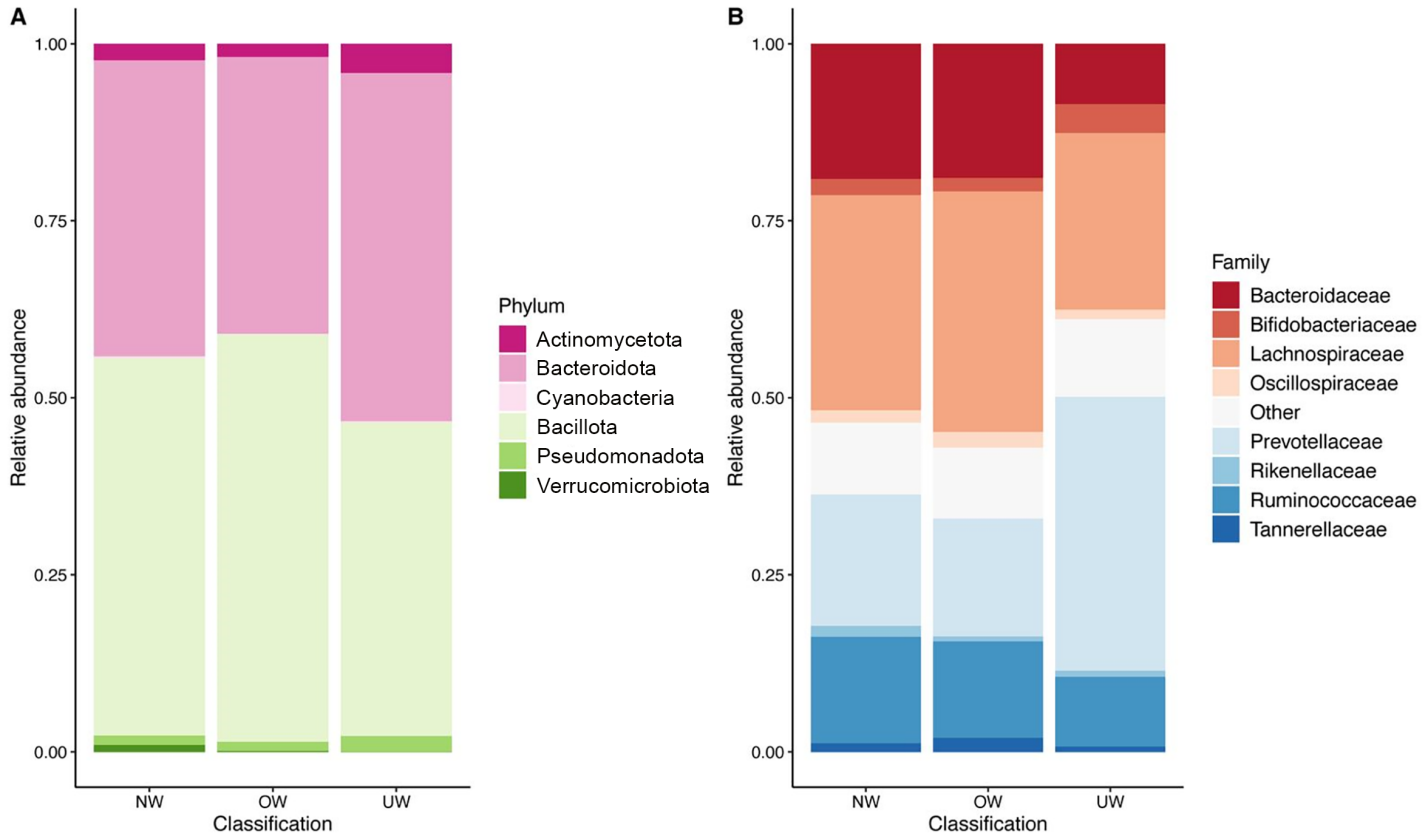
Relative abundances according to BMI categories. (A) Phyla; (B) Family. There is no clear relationship observed between BMI and the phylum-level composition of the microbiome. Although graphical differences are observed in the abundances of the phylo proteobacteria and in the family prevotellaceae, no statistical test showed that these differences were significant. Each row shows the relative abundance of major gut bacterial phyla (A) or family (B) in an individual according to BMI. Created with R (QIIME 2R) package phyloseq (functionplot).

**Figure 5:**
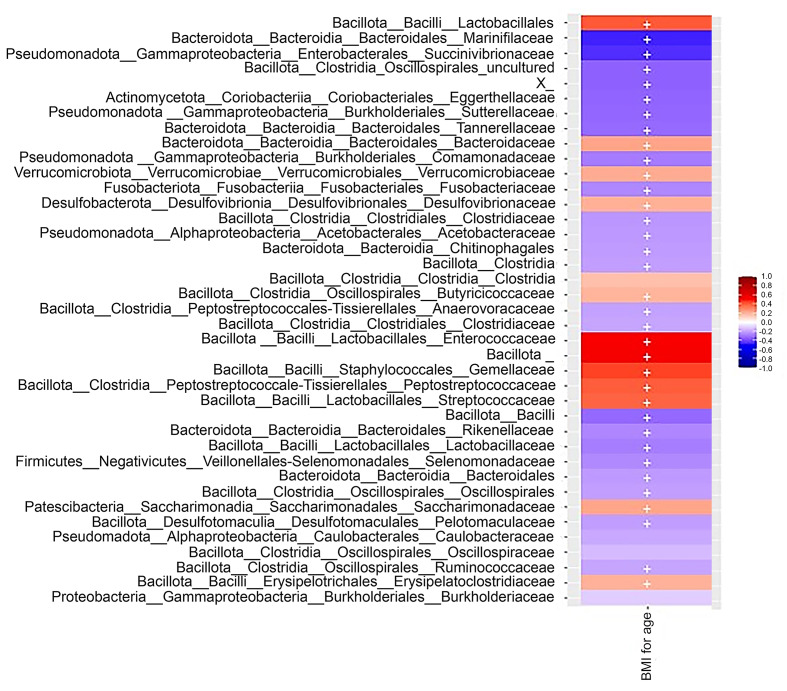
Heatmap of the Spearman correlation between bacterial abundance and the overweight children’s BMI. Positive associations are represented in red, and negative correlations are depicted in blue. Created with QIIME 2.

**Figure 6:**
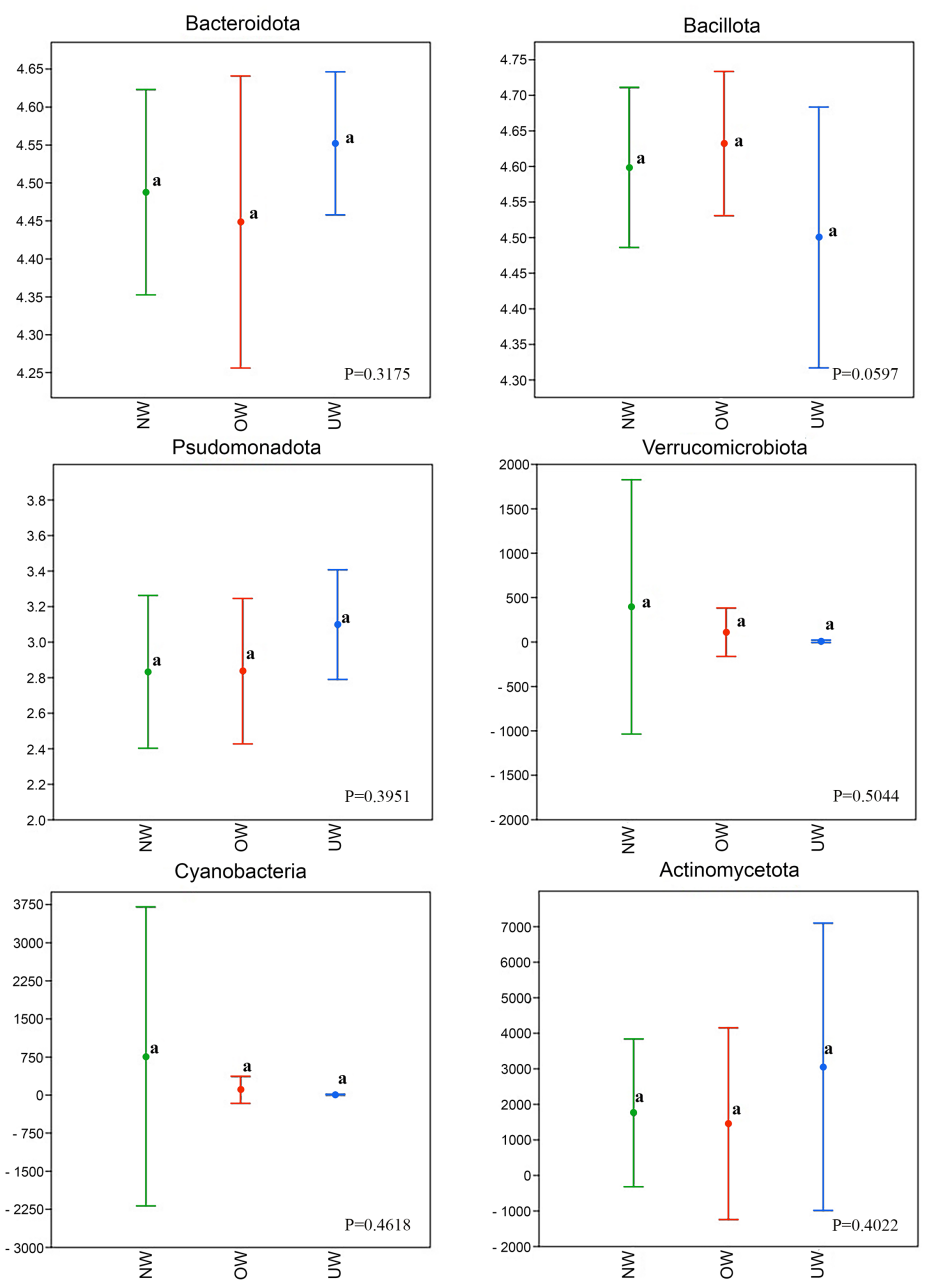
Relative abundance of the predominant Phyla in stool samples of children according to BMI. The box plot illustrates the distribution of profile similarities among taxa within a phylum, along with their standard deviation. In the Y axis, the abundances centered log-ratio (CLR). The superscript letter (a) indicates that there are no significant differences. Created with R (QIIME 2R) package phyloseq (pluginq2-longitudinal).

To understand the relation between faecal microbiota and BMI, phylum relative abundance was calculated (Fig. 4), this yielded six main phyla with varying percentages: Bacillota (47.42%), Bacteroidota (36.75%), Actinomycetota (2.0%), Pseudomonadota (1.21%), Verrucomicrobiota (0.59%), and Cyanobacteria (0.16%). In a heat map representing the abundances of bacterial taxa (scaled from −1 to 1), a greater interaction with the phylum Bacillota was identified, with consistent trends observed independently of BMI and preschool age (Fig. 5). The ANOVA test results showed no significant differences among groups with different BMI (UW, NW, and OW) across any principal phyla (*p* < 0.05); this is also visible in the box plots generated for each phylum analyzed concerning BMI (Fig. 6). The most abundant families were Lachnospiraceae (27.34%), Bacteroidaceae (16.51%), Prevotellaceae (16.36%), Ruminococcaceae (12.40%), Bifidobacteriaceae (1.96%).

In overweight and normal weight children, the Lachnospiraceae family showed greater richness content (24,115.88 and 2,392.02 ASVs, respectively). In contrast, in underweight children, the Prevotellaceae family was represented with 22,562.6 ASVs, and Lachnospiraceae obtained 18,293 ASVs. In normal-weight children, Bacteroidaceae (16,417) and Ruminococcaceae (10,530.32) had greater reads than the other two groups. At this taxonomic level, no statistically significant differences were identified among the groups of children with varying BMIs.

**Figure 7:**
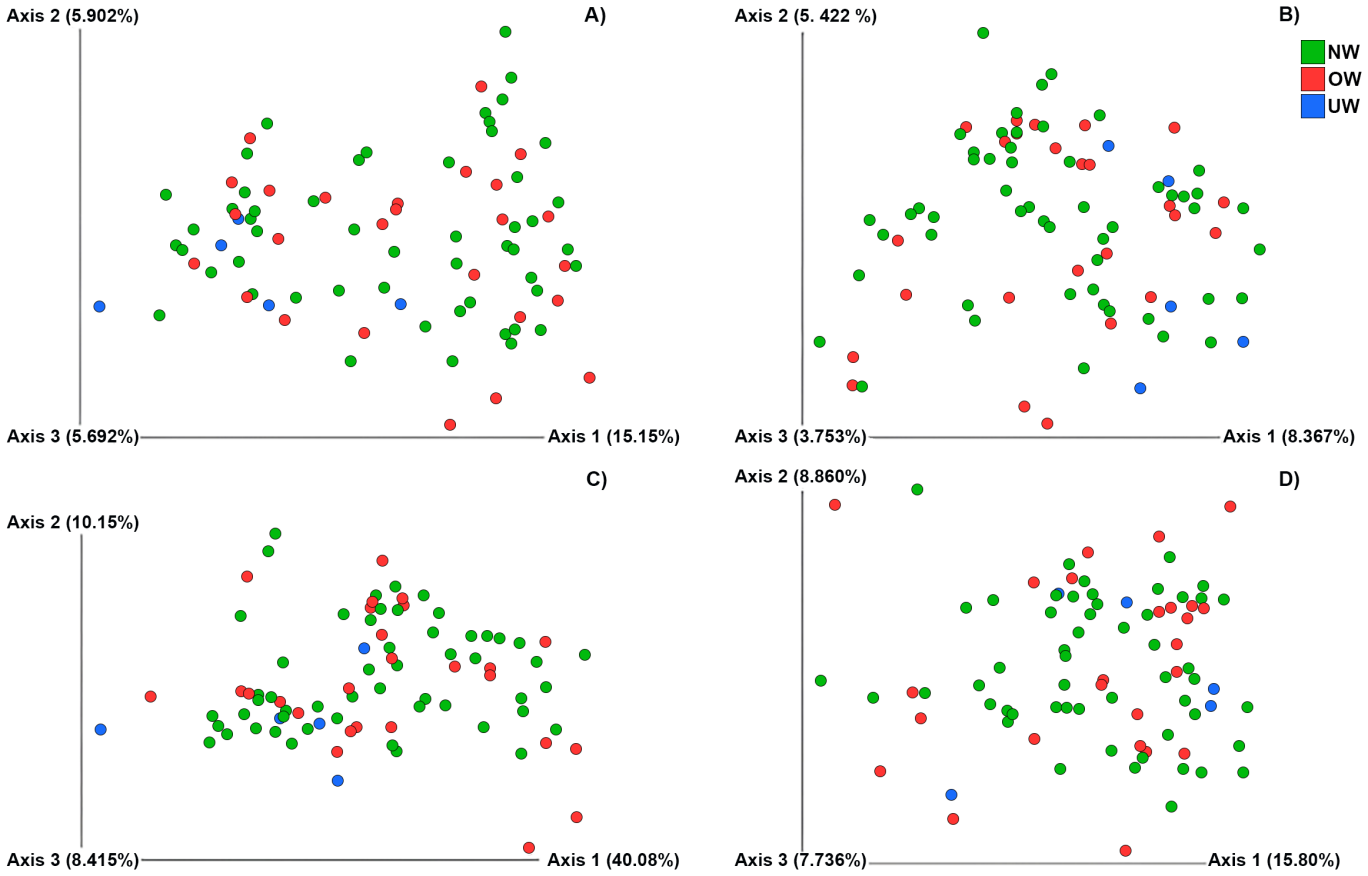
Principal Coordinate Analysis (PCoA) shows Beta-diversity of bacteria in normal weight (NW), overweight (OW) and underweight (UW) children according to BMI. PCoA based on Bray–Curtis (A) and Jaccard (B) dissimilarity was performed with the observation showing no significant differences in gut microbial community structure (*p* = 0.872). In unweighted (C) and weighted (D) analyses, the groupings and distances between the three groups show no structure separation between the analyzed groups. Overweight children are shown in red, normal weight children in green, and underweight children in blue. The analyses were generated by the “qiime diversity adonis” QIIME2 plugin and the *p*-values were calculated using the ADONIS permutation-based statistical test. Created with QIIME 2, plugin Emperor (qiime emperor plot).

Also, no differences were detected in the microbial community structure among the children’s groups (NW, OW, and UW), based on both unweighted and weighted UniFrac distance matrices (weighted UniFrac: Pseudo *F* = 10.50, *R*^2^ = 0.43, *p* = 0.84; unweighted UniFrac: Pseudo *F* = 2.78, *R*^2^ = 0.17, *p* = 0.96). PCoA plots showed that microbial communities were not clustered according to BMI. This pattern was consistent in both metrics (weighted and unweighted UniFrac) (Fig. 7).

## Discussion

The preschool and school community is by far the least studied (Derrien, Alvarez & De Vos, 2019). This study examined 84 preschool children from underweight (UW), normal weight (NW), and overweight (OW), focusing on retrospective clinical data on perinatal and postnatal early factor exposures by the children, to explore patterns with their current body mass index, diet and their implications for the gut microbiota.

The results showed did not show a clear trend that allows establishing a relationship between BMI and delivery type, skin-to-skin contact, hospitalization at birth, or incubator use, suggesting a low likelihood of association between these clinical factors and weight in the preschool children. The type of feeding (breastfeeding, infant formula and mixed) until six months of age was also analyzed in accordance with current BMI of preschoolers. These analyses showed a clustering between normal weight and children with exclusive breastfeeding and, to a lesser extent, mixed feeding (Fig. 1). In general, exclusive breastfeeding up to 6 months of age has been associated with better long-term health status for children (Froń & Orczyk-Pawiłowicz, 2024; Horta & Victora, 2013). However, the role of exclusive breastfeeding in children’s weight is not yet entirely clear. In this regard, authors such as Mantzorou et al. (2022) associated breastfeeding with a lower risk of childhood obesity, while other studies did not support significant associations between breastfeeding and childhood overweight prevention and obesity, especially when considering other factors (Durmuş et al., 2011; Huus et al., 2008). Formula feeding has previously been reported as a risk factor for developing overweight in childhood (Dharod et al., 2023); we also found a grouping between formula feeding and the overweight category in preschool children, contrary to another study that has reported that exclusive formula feeding could be a risk factor for low birth weight in children at ages 3 and 5 (Li et al., 2022). These discrepancies among the different studies may be ascribed to the inclusion of other maternal factors, some of which were not considered in this work. When low birth weight has been specifically evaluated relative to its association with childhood overweight, the results have produced conflicting findings (Andriani, 2021; Grillo, Mariani & Ferraris, 2022). In this study, we don’t see a clear influence, since all birth weight categories were clustering with children with NW, except those with very low birth weight (<1.5 kg), who tended to be underweight at the time of the evaluation.

Beyond the obtained results, we also recognize the need to broaden this perspective by including other circumstances occurring during gestational development, such as tobacco use, pregnancy diet, and socioeconomic status, as well as current factors in the child’s environment, such as family eating habits, physical activity, screen time, and sleep patterns. Together, all these factors could play a key role in understanding their impact on BMI and the child’s gut microbiota (Levin et al., 2016; Peng et al., 2024; Wang et al., 2022). Although the inclusion of additional variables in the study could influence the results, our findings may also suggest that during the preschool years, the impact of certain perinatal and postnatal exposures on BMI and consequently on gut microbiota composition could be attenuated.

Given the exploratory nature of microbiota studies based on 16S rRNA gene sequencing, the study was designed to characterize overall microbial community structure rather than to ensure balanced group sizes. Alpha and beta diversity analyses were performed using QIIME2, which are commonly applied in observational studies with naturally occurring group sizes. However, the small number of underweight participants may limit statistical power for detecting differences involving this group; therefore, results related to underweight children should be interpreted with caution.

Regarding the assessment of the gut microbiota, the high levels of alpha diversity observed in this study were more pronounced in the normal-weight group, a pattern generally interpreted as an indicator of a healthier gut ecosystem. However, these values appear unusually elevated considering the overall low dietary quality of the children. Diet is known to influence gut microbial diversity, although the magnitude and direction of these associations vary. Several studies have emphasized the importance of specific food sources, in that sense, Herman et al. (2020) reported that while most animal-derived foods showed weak associations with microbiota structure, protein-rich animal products such as meats were positively correlated with microbial diversity as measured by the Shannon index. Notably, when total protein intake was analyzed as a macronutrient, no correlation with alpha-diversity metrics was observed, likely because this category combines plant- and animal-derived proteins.

These findings are consistent with the scientific literature showing effects on microbial composition associated not only protein quantity but also that protein source has similar or even stronger effects (Blakeley-Ruiz et al., 2025), although evidence across populations remains heterogeneous. For example, Clarke et al. (2014) reported higher diversity in individuals consuming protein-rich diets, whereas comparative studies of Western *versus* rural African populations have shown that diets high in animal protein and fat but low in plant fiber may be linked to lower overall diversity (De Filippo et al., 2010). Govindan et al. (2025) conducted a study in adults undergoing plant protein *vs* animal protein interventions for 12 weeks, demonstrating that participants with plant protein experienced greater microbial diversity and greater abundance of short-chain fatty acid-producing bacteria. Thus, current evidence suggests that the source of dietary protein, rather than total protein intake, plays a critical role in shaping gut microbial diversity, and this may provide an explanation for the lack of correlation between total protein intake and the alpha diversity values observed in the present study.

Additionally, Herman et al. (2020) noted that the consumption of fortified, non-whole foods may also influence microbiota composition, adding further complexity to the relationship between dietary patterns and gut microbial diversity in children.

Although high bacterial richness and diversity are generally associated with balanced diets, it is important to consider that microbiota composition is influenced by a multitude of factors beyond immediate diet, including genetic and environmental factors, and potentially others not yet studied. Therefore, diversity does not necessarily reflect diet quality or an individual’s body condition. Williams, Hammer & Williams (2024) observed that while a diverse microbiota is often considered beneficial, diversity alone does not consistently mean better host health, since diversification can sometimes reflect a loss of functional homogeneity. Moreover, Williams, Hammer & Williams (2024) suggest that there is no clear link between alpha diversity and health, either within or between species.

Concerning gut microbial taxa, the results showed that OW children had no difference compared to NW or UW children. Bacillota and Bacteroidota were found to be the predominant taxa, coinciding with previous reports (Ma et al., 2023). Nevertheless, a difference in the abundance of this phylum between OW, NW and UW children was not found. Similar results at the phylum level have been observed in another work with Mexican school-aged children (López-Contreras et al., 2018). Conversely, in the study by Shin & Cho (2020), the relative abundance of the Bacteroidota phylum was lower in the obese group than in the normal-weight group, and this decrease was negatively correlated with body mass index (BMI). A study conducted on Mexican children between 5 and 11 years old, also showed that the characterization of microbial diversity and abundance of bacterial communities, in general, was similar between normal weight and obese children, although it does point out differences at lower taxonomic levels (family and genera), especially in the group of children aged 9 to 11 years (Maya-Lucas et al., 2019).

Here, the observed differences between groups of children with different BMIs (although they did not show statistical significance) may reflect the role of certain bacterial families such as Prevotellaceae and Lachnospiraceae. Some other studies have emphasized the relationship between the abundances of some bacterial families and their association with BMI of children (Aguilar et al., 2020; Chávez-Carbajal et al., 2019; Salonen et al., 2014). In particular, the Prevotellaceae family was found to be the most important bacterial family associated with BMI. Children with obesity and high waist/height ratio had lower Bacteroidaceae and Prevotellaceae, and higher abundance of Lactobacillaceae compared to normal-weight children (Aguilar et al., 2020).

Although these results are like those obtained in this study, it appears that Prevotellaceae has a more complex background, as the results can be highly variable when associated with other factors. For example, Cuevas-Sierra et al. (2020), associated the abundance of Prevotellaceae with BMI in adult women, finding that higher Prevotellaceae abundance and higher genetic risk indexes led to more obesity in women, compared to women with higher genetic risk and lower Prevotellaceae abundance.

While Lachnospiraceae are generally considered beneficial bacteria, they have been associated with metabolic diseases when their abundance increases, likely due to the production of short-chain fatty acids (SCFAs) other than butyrate. High abundances of Lachnospiraceae positively correlated with glucose and/or lipid metabolism, indicating a capacity to cause metabolic disturbance (Vacca et al., 2020).

Emerging evidence has indicated the pathological effects of specific SCFAs, such as acetate and propionate, in various metabolic disorders, including obesity (Chávez-Carbajal et al., 2019; Salonen et al., 2014). These studies have reported significant increases in the Lachnospiraceae family; however, they have been conducted in adult individuals where the weight range between lean and obese individuals is greater.

On the other hand, sexual differentiation has been considered a factor in explaining variations in microbiota (Bergström et al., 2014; Markle et al., 2013). This is directly related to the brain-gut axis and hormone interaction, given that males and females exhibit sexually dimorphic patterns in energy and nutritional requirements across the lifespan (Markle et al., 2013). We considered sex as a factor in analyzing the microbiota of preschool children, but no significative differences were detected. In line with previous reports, childhood is characterized by gonadal hormone quiescence and minimal sex-specific development (Jašarević, Morrison & Bale, 2016), which could explain the absence of sex related differences in children gut microbiota composition.

Likewise, the sample size and the homogeneity of the group studied towards normal weight could have limited our ability to detect significant microbial differences between BMI categories. Nonetheless, we consider that the 84 independent biological replicates are comparable to similar studies in pediatric populations (Jayasinghe et al., 2020). Therefore, the lack of significant differences may suggest the need to reconsider the role of BMI as a determining factor in shaping the gut microbiota at this developmental stage. Moreover, as the gut microbiota is still developing in early childhood, clear associations with BMI may not emerge until microbial composition becomes more stable. Even so, studies with larger sample sizes and a longitudinal design in different regions are needed to provide a more comprehensive understanding of these associations.

Hence, this study has several limitations that should be acknowledged. First, these findings are not generalized to all preschool children and may varyin other geographic regions or cultural contexts. Second, the study followed a cross-sectional design and did not perform a priori sample size calculation, generating unequal sample sizes across groups, mainly a small number of underweight participants; hence, results related to this should be interpreted with caution, including perinatal and postnatal factors, current BMI, diet, and gut microbiota composition. Finally, although children with antibiotic use were excluded and clinical history were collected to confirm the absence of acute infections and during the month prior to fecal sampling, the use of topical antimicrobial treatments was not recorded and therefore cannot be completely excluded. Nevertheless, the same geographic area of the study population, clinical method, and laboratory tests reinforce the internal validity of the study and provide a solid base for similar populations.

## Conclusions

In conclusion, our results indicate a grouping between exclusive breastfeeding and normal weight in preschool children. Birth weight and premature birth also appear to influence BMI in these children. Regarding gut microbiota, Bacillota and Bacteroidota were predominant bacterial phyla across all BMI groups. Although Prevotellaceae was more abundant in underweight children and Lachnospiraceae in those with higher BMI, these trends were not statistically significant and warrant further investigation, as multiple interacting factors cannot be fully investigated within a single study. Furthermore, the influence of dietary patterns and microbiota composition introduces additional layers of complexity to this relationship. Although high bacterial richness and diversity are generally associated with balanced diets, it is important to consider that microbiota composition is influenced by a multitude of factors beyond immediate diet and nutritional status, including genetic and environmental factors, and potentially others not yet studied. Finally, longitudinal research at different stages of childhood, integrating extensive clinical and lifestyle data, is essential to understanding the extent to which these influence the development and establishment of the gut microbiota and, consequently, children’s weight. This knowledge will support the design of preventive strategies aimed at controlling factors that could contribute to unhealthy weight in childhood.

## Supplemental Information

10.7717/peerj.21253/supp-1 Supplemental Information 1 Body Mass Index BMI was calculated as weight (kg) divided by height squared (m^2^) and classified according to the World Health Organization (WHO) growth reference standards for age and sex

10.7717/peerj.21253/supp-2 Supplemental Information 2 Calories consumption by food group a Kruskall Wallis test for comparison between BMI categories.

10.7717/peerj.21253/supp-3 Supplemental Information 3 ASVs of the main phyla Bacterial composition of faecal samples from preschool-aged children classified according to Body Mass Index (BMI) category and sex. Values represent the relative abundance of the predominant bacterial phyla identified by 16S rRNA gene sequencing

10.7717/peerj.21253/supp-4 Supplemental Information 4 STROBE checklist Checklist of items recommended for reporting cross-sectional studies according to the STROBE (Strengthening the Reporting of Observational Studies in Epidemiology) guidelines.

10.7717/peerj.21253/supp-5 Supplemental Information 5 Metadata of faecal samples included in the study Sample identification, collection date, environmental context, geographical location, and host characteristics such as age, sex, height, body mass index (BMI), and body mass

10.7717/peerj.21253/supp-6 Supplemental Information 6 Sample identification metadata according to BioProject and BioSample, as well as library characteristics, instrument model, and FASTA file

## Additional Information and Declarations

### Data Availability

The following information was supplied regarding data availability: The data is available at Figshare: Hernandez-Rodriguez, Dolores (2025). Sequence Data. Faecal microbiome composition concerning body mass index and early-life factors in Mexican preschool-aged children. figshare. Dataset. https://doi.org/10.6084/m9.figshare.30290248.v1
The raw sequence data are available at NCBI BioProject: PRJNA1337777.
