# Supplementary material for: Fecal microbiota composition concerning body mass index and early-life factors in Mexican preschool-aged children: a cross-sectional study
Source: PeerJ. 2026 Jun 3;14:e21253. doi: 10.7717/peerj.21253 (PMC13242193; doi:10.7717/peerj.21253)
Supplement: Supplemental Information 1 — BMI was calculated as weight (kg) divided by height squared (m2) and classified according to the World Health Organization (WHO) growth reference standards for age and sex [file peerj-14-21253-s001.docx]

**Suppl Table S1. Body Mass Index**

| **Sex** | **Age** | **Weight (Kg)** | **Size (cm)** | **BMI Kg/m2** | **BMI (classification)** |
| --- | --- | --- | --- | --- | --- |
| Female | 5 | 16.70 | 107 | 14.53 | NW |
| Female | 5 | 19.00 | 108 | 16.44 | NW |
| Female | 4 | 15.40 | 103 | 14.60 | NW |
| Female | 4 | 13.60 | 97 | 14.45 | NW |
| Female | 3 | 15.20 | 100 | 15.35 | NW |
| Female | 4 | 17.10 | 106 | 15.28 | NW |
| Female | 4 | 15.90 | 100 | 15.77 | NW |
| Female | 4 | 18.40 | 110 | 15.12 | NW |
| Female | 5 | 17.10 | 105 | 15.51 | NW |
| Female | 5 | 14.70 | 102 | 14.18 | NW |
| Female | 5 | 17.10 | 109 | 14.37 | NW |
| Female | 4 | 17.30 | 103 | 16.24 | NW |
| Female | 5 | 18.2 | 110 | 15.04 | NW |
| Female | 4 | 17.10 | 103 | 16.12 | NW |
| Female | 5 | 19.30 | 109 | 16.16 | NW |
| Female | 4 | 16.40 | 106 | 14.49 | NW |
| Female | 5 | 19.00 | 111 | 15.45 | NW |
| Female | 4 | 16.50 | 102 | 15.86 | NW |
| Female | 3 | 14.60 | 99 | 15.05 | NW |
| Female | 5 | 16.40 | 105 | 14.88 | NW |
| Female | 4 | 17.50 | 104 | 16.34 | NW |
| Female | 4 | 21.10 | 116 | 15.68 | NW |
| Female | 3 | 14.6 | 99 | 15.05 | NW |
| Female | 4 | 21.90 | 108 | 18.71 | OW |
| Female | 5 | 22.50 | 108 | 19.22 | OW |
| Female | 5 | 25.50 | 114 | 19.48 | OW |
| Female | 3 | *18.70* | *103* | *17.52* | *OW* |
| Female | 4 | 19.10 | 102 | 18.43 | OW |
| Female | 4 | 21.60 | 112 | 17.37 | OW |
| Female | 4 | 19.30 | 107 | 17.02 | OW |
| Female | 6 | 27.10 | 122 | 18.21 | OW |
| Female | 5 | 18.30 | 104 | 16.92 | OW |
| Female | 5 | 22.50 | 115 | 16.90 | OW |
| Female | 5 | 15.30 | 105 | 13.82 | UW |
| Female | 4 | 16.30 | 115 | 12.33 | UW |
| Female | 3 | 14.60 | 103 | 13.66 | UW |
| Male | 4 | 18.60 | 107 | 16.25 | NW |
| Male | 4 | 17.10 | 103 | 16.28 | NW |
| Male | 5 | 15.40 | 104 | 14.24 | NW |
| Male | 4 | 19.00 | 111 | 15.42 | NW |
| Male | 5 | 17.50 | 107 | 15.43 | NW |
| Male | 4 | 16.30 | 103 | 15.36 | NW |
| Male | 5 | 17.30 | 107 | 15.00 | NW |
| Male | 5 | 15.50 | 105 | 14.06 | NW |
| Male | 5 | 20.40 | 119 | 14.50 | NW |
| Male | 3 | 17.40 | 106 | 15.60 | NW |
| Male | 4 | 17.10 | 104.3 | 15.72 | NW |
| Male | 4 | 15.90 | 104 | 14.70 | NW |
| Male | 4 | 17.50 | 111 | 14.20 | NW |
| Male | 4 | 17.20 | 104 | 15.93 | NW |
| Male | 5 | 17.90 | 112 | 14.19 | NW |
| Male | 4 | 20.40 | 112 | 16.38 | NW |
| Male | 4 | 16.30 | 101 | 16.14 | NW |
| Male | 4 | 16.90 | 106 | 15.04 | NW |
| Male | 5 | 18.90 | 110 | 15.51 | NW |
| Male | 6 | 18.90 | 111 | 15.48 | NW |
| Male | 5 | 15.10 | 102 | 14.57 | NW |
| Male | 4 | 17.00 | 105 | 15.57 | NW |
| Male | 5 | 19.00 | 111 | 15.50 | NW |
| Male | 4 | 17.30 | 105 | 15.81 | NW |
| Male | 5 | 18.10 | 110 | 14.96 | NW |
| Male | 5 | 19.10 | 110 | 15.93 | NW |
| Male | 3 | 15.40 | 103 | 14.66 | NW |
| Male | 5 | 19.10 | 118 | 13.81 | NW |
| Male | 6 | 19.70 | 114 | 15.05 | NW |
| Male | 4 | 20.30 | 117 | 14.96 | NW |
| Male | 4 | 16.20 | 102 | 15.60 | NW |
| Male | 2 | 12.70 | 86.5 | 16.97 | OW |
| Male | 5 | 21.80 | 111 | 17.73 | OW |
| Male | 4 | 20.80 | 108 | 17.80 | OW |
| Male | 4 | 20.00 | 107 | 17.50 | OW |
| Male | 5 | 27.80 | 122 | 18.56 | OW |
| Male | 5 | 22.10 | 115 | 16.74 | OW |
| Male | 4 | 19.20 | 105 | 17.45 | OW |
| Male | 5 | 23.90 | 116 | 17.76 | OW |
| Male | 4 | 17.40 | 102 | 16.89 | OW |
| Male | 3 | 16.60 | 99 | 16.90 | OW |
| Male | 4 | 19.20 | 107 | 16.90 | OW |
| Male | 5 | 25.90 | 118 | 18.54 | OW |
| Male | 4 | 20.70 | 109 | 17.42 | OW |
| Male | 5 | 21.50 | 112 | 17.14 | OW |
| Male | 4 | 24.50 | 110 | 20.10 | OW |
| Male | 5 | 13.50 | 103 | 12.63 | UW |
| Male | 5 | 16.60 | 112 | 13.30 | UW |

BMI was calculated as weight (kg) divided by height squared (m²) and classified according to the World Health Organization (WHO) growth reference standards for age and sex.
