# Supplementary material for: Fecal microbiota composition concerning body mass index and early-life factors in Mexican preschool-aged children: a cross-sectional study
Source: PeerJ. 2026 Jun 3;14:e21253. doi: 10.7717/peerj.21253 (PMC13242193; doi:10.7717/peerj.21253)
Supplement: Supplemental Information 2 — a Kruskall Wallis test for comparison between BMI categories. [file peerj-14-21253-s002.docx]

**Supplementary Table S2. Calorie consumption by food group**

| **Food group** | **Median** | **Range** | **Min** | **Max** | **Percentiles** | | | ***p*-value^a^** |
| --- | --- | --- | --- | --- | --- | --- | --- | --- |
|  |  |  |  |  | **25** | **50** | **75** |  |
| Cereals | 317.9 | 1541.1 | 2.87 | 1543.97 | 71.1 | 317.9 | 410.3 | 0.328 |
| Legumes | 7.8 | 47.9 | 0.0 | 47.9 | 3.5 | 7.8 | 15.57 | 0.594 |
| Vegetables | 2.9 | 17.3 | 0.0 | 17.37 | 2.23 | 2.97 | 5.5 | 0.29 |
| Fruits | 8.8 | 177.9 | 0.0 | 177.9 | 4.9 | 8.83 | 12.03 | 0.14 |
| Dairy | 12.8 | 37.3 | 0.64 | 37.97 | 9.27 | 12.83 | 17.33 | 0.31 |
| Meat | 7.6 | 28.1 | 0.34 | 28.5 | 4.93 | 7.67 | 11.1 | 0.57 |
| Fish | 0.13 | 15.8 | 0.0 | 15.87 | 0.0 | 0.13 | 0.64 | 0.17 |
| Eggs | 1.09 | 5.43 | 0.0 | 5.4 | 1.09 | 1.09 | 2.27 | 0.4 |
| Cakes | 8.2 | 108.9 | 0.0 | 108.9 | 2.74 | 8.2 | 16.1 | 0.35 |
| Oils | 3 | 17.9 | 0.0 | 17.97 | 2.50 | 3.0 | 6.0 | 0.39 |
| Sugary drinks | 625.47 | 5,600.1 | 0.0 | 5600.17 | 373.67 | 625.47 | 945.5 | 0.18 |
| Processed food | 693.3 | 4,702.5 | 0.0 | 4702.5 | 250.1 | 693.3 | 1,355.9 | 0.23 |
| Snacks | 3.8 | 28.7 | 0.0 | 28.73 | 0.96 | 3.83 | 11.5 | 0.77 |
| Condiments | 0.32 | 2.36 | 0.0 | 2.36 | 0.08 | 0.32 | 0.95 | 0.035 |
| ^a^Kruskall Wallis test for comparison between BMI categories. | | | | | | | | |
