# Supplementary material for: Fecal microbiota composition concerning body mass index and early-life factors in Mexican preschool-aged children: a cross-sectional study
Source: PeerJ. 2026 Jun 3;14:e21253. doi: 10.7717/peerj.21253 (PMC13242193; doi:10.7717/peerj.21253)
Supplement: Supplemental Information 3 — Bacterial composition of faecal samples from preschool-aged children classified according to Body Mass Index (BMI) category and sex. Values represent the relative abundance of the predominant bacterial phyla identified by 16S rRNA gene sequencing [file peerj-14-21253-s003.docx]

| **ID sample** | **Bacillota** | **Bacteroidota** | **Acinomycetota** | **Pseudomonadota** | **Verrucomicrobiota** | **Cyanobacteria** | **BMI Classification** | **Child Sex** |
| --- | --- | --- | --- | --- | --- | --- | --- | --- |
| **S71** | 44693 | 30470 | 7944 | 1232 | 0 | 1555 | NW | Female |
| **S23** | 30923 | 32359 | 666 | 562 | 0 | 546 | NW | Female |
| **S84** | 43037 | 26488 | 134 | 356 | 0 | 0 | NW | Female |
| **S55** | 50301 | 33340 | 3080 | 4579 | 0 | 50 | NW | Female |
| **S76** | 61599 | 21714 | 4599 | 658 | 150 | 0 | NW | Female |
| **S42** | 28604 | 26530 | 592 | 411 | 70 | 0 | NW | Female |
| **S24** | 32443 | 33265 | 6687 | 1445 | 0 | 0 | NW | Female |
| **S58** | 34426 | 27256 | 1166 | 1814 | 33 | 0 | NW | Female |
| **S45** | 46228 | 10403 | 453 | 228 | 0 | 0 | NW | Female |
| **S35** | 44232 | 30604 | 4191 | 1314 | 0 | 0 | NW | Female |
| **S69** | 40190 | 25149 | 411 | 203 | 255 | 0 | NW | Female |
| **S47** | 41407 | 26286 | 9848 | 804 | 0 | 0 | NW | Female |
| **S37** | 53364 | 44066 | 12418 | 2270 | 0 | 0 | NW | Female |
| **S31** | 58660 | 38113 | 37 | 965 | 74 | 0 | NW | Female |
| **S82** | 47019 | 30667 | 1777 | 1643 | 0 | 328 | NW | Female |
| **S53** | 36845 | 28218 | 501 | 1782 | 0 | 406 | NW | Female |
| **S62** | 48547 | 38954 | 731 | 176 | 0 | 0 | NW | Female |
| **S77** | 44953 | 27520 | 626 | 4033 | 0 | 0 | NW | Female |
| **S88** | 32977 | 39755 | 0 | 979 | 0 | 0 | NW | Female |
| **S15** | 36081 | 17990 | 5358 | 413 | 0 | 0 | NW | Female |
| **S56** | 31043 | 39540 | 2962 | 1347 | 0 | 0 | NW | Female |
| **S78** | 29780 | 24904 | 4255 | 350 | 8490 | 388 | NW | Female |
| **S91** | 34040 | 24897 | 1529 | 1169 | 28 | 0 | NW | Female |
| **S54** | 39934 | 37133 | 5047 | 70 | 12 | 0 | OW | Female |
| **S11** | 44962 | 32680 | 213 | 884 | 623 | 933 | OW | Female |
| **S41** | 36540 | 14543 | 0 | 320 | 0 | 0 | OW | Female |
| **S8** | 37099 | 41096 | 211 | 1244 | 0 | 84 | OW | Female |
| **S3** | 53875 | 40448 | 235 | 537 | 0 | 0 | OW | Female |
| **S51** | 46582 | 25035 | 33 | 521 | 0 | 0 | OW | Female |
| **S73** | 48692 | 25934 | 514 | 1211 | 0 | 241 | OW | Female |
| **S17** | 22642 | 34865 | 2318 | 2265 | 831 | 0 | OW | Female |
| **S75** | 39526 | 19878 | 204 | 91 | 0 | 74 | OW | Female |
| **S20** | 29281 | 28865 | 457 | 739 | 678 | 785 | OW | Female |
| **S50** | 35428 | 17972 | 249 | 366 | 19139 | 0 | UW | Female |
| **S63** | 35313 | 30111 | 6981 | 437 | 0 | 0 | UW | Female |
| **S7** | 15067 | 24869 | 2129 | 53 | 0 | 0 | UW | Female |
| **S43** | 31853 | 55970 | 162 | 525 | 1007 | 0 | NW | Male |
| **S72** | 29193 | 33921 | 772 | 463 | 0 | 0 | NW | Male |
| **S57** | 39263 | 8590 | 43 | 180 | 0 | 0 | NW | Male |
| **S39** | 55262 | 32097 | 731 | 894 | 0 | 0 | NW | Male |
| **S44** | 42365 | 27954 | 233 | 703 | 0 | 88 | NW | Male |
| **S80** | 24035 | 40140 | 1878 | 335 | 0 | 1351 | NW | Male |
| **S70** | 28690 | 35486 | 762 | 662 | 0 | 0 | NW | Male |
| **S46** | 28714 | 29260 | 63 | 354 | 839 | 234 | NW | Male |
| **S12** | 55181 | 37136 | 27 | 1041 | 0 | 0 | NW | Male |
| **S9** | 37653 | 39594 | 3526 | 1464 | 0 | 372 | NW | Male |
| **S89** | 34491 | 39755 | 0 | 979 | 0 | 0 | NW | Male |
| **S61** | 39229 | 23586 | 614 | 2553 | 1352 | 836 | NW | Male |
| **S29** | 27146 | 26488 | 41 | 1186 | 0 | 32 | NW | Male |
| **S81** | 65735 | 26275 | 136 | 556 | 0 | 24 | NW | Male |
| **S14** | 32582 | 42769 | 397 | 1174 | 62 | 0 | NW | Male |
| **S66** | 43732 | 33845 | 284 | 852 | 0 | 0 | NW | Male |
| **S79** | 23660 | 26356 | 21 | 153 | 0 | 474 | NW | Male |
| **S1** |  |  |  |  |  |  | NW | Male |
| **S16** | 50182 | 45649 | 310 | 565 | 0 | 0 | NW | Male |
| **S28** | 53318 | 21648 | 637 | 1592 | 0 | 0 | NW | Male |
| **S49** | 47346 | 43779 | 96 | 3706 | 0 | 0 | NW | Male |
| **S32** | 40603 | 24933 | 2699 | 656 | 0 | 0 | NW | Male |
| **S18** | 57250 | 33040 | 3629 | 642 | 0 | 0 | NW | Male |
| **S65** | 52334 | 40056 | 0 | 149 | 1833 | 0 | NW | Male |
| **S74** | 57193 | 30173 | 323 | 1808 | 0 | 0 | NW | Male |
| **S59** | 48396 | 45173 | 259 | 2303 | 0 | 0 | NW | Male |
| **S19** | 41809 | 43994 | 231 | 776 | 629 | 0 | NW | Male |
| **S21** | 35164 | 53911 | 3144 | 2885 | 129 | 244 | NW | Male |
| **S6** | 34358 | 34482 | 460 | 3387 | 0 | 10 | NW | Male |
| **S33** | 40114 | 39891 | 2502 | 953 | 43 | 0 | NW | Male |
| **S10** | 25885 | 22607 | 2601 | 3307 | 0 | 108 | NW | Male |
| **S25** | 36944 | 30174 | 1998 | 927 | 5990 | 67 | OW | Male |
| **S54** | 39934 | 37133 | 5047 | 70 | 12 | 0 | OW | Male |
| **S2** | 68658 | 44538 | 258 | 1725 | 187 | 0 | OW | Male |
| **S64** | 34503 | 30034 | 3379 | 446 | 3 | 2323 | OW | Male |
| **S26** | 42326 | 38474 | 352 | 352 | 87 | 0 | OW | Male |
| **S5** | 33068 | 10929 | 2881 | 566 | 3 | 48 | OW | Male |
| **S22** | 57137 | 21800 | 5555 | 184 | 0 | 0 | OW | Male |
| **S36** | 48084 | 37444 | 0 | 1116 | 356 | 0 | OW | Male |
| **S90** | 46464 | 15817 | 6902 | 822 | 17 | 0 | OW | Male |
| **S13** | 61933 | 34342 | 2578 | 157 | 79 | 0 | OW | Male |
| **S27** | 34957 | 50392 | 0 | 58 | 0 | 0 | OW | Male |
| **S68** | 45814 | 40767 | 855 | 873 | 6 | 0 | OW | Male |
| **S48** | 44788 | 30737 | 1573 | 570 | 30 | 109 | OW | Male |
| **S67** | 53547 | 42574 | 248 | 989 | 0 | 0 | OW | Male |
| **S30** | 37691 | 29267 | 1155 | 385 | 0 | 0 | OW | Male |
| **S34** | 40442 | 35184 | 521 | 1180 | 0 | 0 | UW | Male |
| **S38** | 41748 | 38753 | 1808 | 417 | 0 | 0 | UW | Male |
