# Supplementary material for: Fecal microbiota composition concerning body mass index and early-life factors in Mexican preschool-aged children: a cross-sectional study
Source: PeerJ. 2026 Jun 3;14:e21253. doi: 10.7717/peerj.21253 (PMC13242193; doi:10.7717/peerj.21253)
Supplement: Supplemental Information 4 — Checklist of items recommended for reporting cross-sectional studies according to the STROBE (Strengthening the Reporting of Observational Studies in Epidemiology) guidelines. [file peerj-14-21253-s004.doc]

STROBE Statement—Checklist of items that should be included in reports of ***cross-sectional studies***

|  | Item No | Recommendation | Wher Where in the manuscript this is addressed |
| --- | --- | --- | --- |
| **Title and abstract** | 1 | (*a*) Indicate the study’s design with a commonly used term in the title or the abstract | Line 3 |
|  |  | (*b*) Provide in the abstract an informative and balanced summary of what was done and what was found | Lines 23-42 |
| Introduction | | |  |
| Background/rationale | 2 | Explain the scientific background and rationale for the investigation being reported | Introduction, lines 45-80 |
| Objectives | 3 | State specific objectives, including any prespecified hypotheses | Introduction, last paragraph lines 81-87 |
| Methods | | |  |
| Study design | 4 | Present key elements of study design early in the paper | Line 92 |
| Setting | 5 | Describe the setting, locations, and relevant dates, including periods of recruitment, exposure, follow-up, and data collection | Lines 92-93, 118-120 |
| Participants | 6 | (*a*) Give the eligibility criteria, and the sources and methods of selection of participants | Lines 98-99 |
| Variables | 7 | Clearly define all outcomes, exposures, predictors, potential confounders, and effect modifiers. Give diagnostic criteria, if applicable | Clinical data lines 124-132  BMI scores lines-139-141 |
| Data sources/ measurement | 8* | For each variable of interest, give sources of data and details of methods of assessment (measurement). Describe comparability of assessment methods if there is more than one group | Clinical data lines 120-123  BMI Lines 135-136  RNA Analysis 144-180 |
| Bias | 9 | Describe any efforts to address potential sources of bias | Lines 86-87, 159-160 |
| Study size | 10 | Explain how the study size was arrived at | Lines 95-97 |
| Quantitative variables | 11 | Explain how quantitative variables were handled in the analyses. If applicable, describe which groupings were chosen and why | BMI lines 140-141  16 S RNA 158-180  Statistical analysis 183-192 |
| Statistical methods | 12 | (*a*) Describe all statistical methods, including those used to control for confounding | Lines 183-192 |
|  |  | (*b*) Describe any methods used to examine subgroups and interactions | Lines 191-192 |
|  |  | (*c*) Explain how missing data were addressed | All analyses used a **complete** approach |
|  |  | (*d*) If applicable, describe analytical methods taking account of sampling strategy | Lines 170-171 |
|  |  | (*e*) Describe any sensitivity analyses | Lines 164-167 |
| Results | | |  |
| Participants | 13* | (a) Report numbers of individuals at each stage of study—eg numbers potentially eligible, examined for eligibility, confirmed eligible, included in the study, completing follow-up, and analysed | Lines- 95-97. Results line 199-200 |
|  |  | (b) Give reasons for non-participation at each stage | Lines 95-97. Results line 199-200 |
|  |  | (c) Consider use of a flow diagram | N/A |
| Descriptive data | 14* | (a) Give characteristics of study participants (eg demographic, clinical, social) and information on exposures and potential confounders | Lines 199-206 |
|  |  | (b) Indicate number of participants with missing data for each variable of interest | N/A |
| Outcome data | 15* | Report numbers of outcome events or summary measures | Lines 198-249 |
| Main results | 16 | (*a*) Give unadjusted estimates and, if applicable, confounder-adjusted estimates and their precision (eg, 95% confidence interval). Make clear which confounders were adjusted for and why they were included | N/A |
|  |  | (*b*) Report category boundaries when continuous variables were categorized | Lines 125-132, 140-141 |
|  |  | (*c*) If relevant, consider translating estimates of relative risk into absolute risk for a meaningful time period | N/A |
| Other analyses | 17 | Report other analyses done—eg analyses of subgroups and interactions, and sensitivity analyses | No formal subgroup or interaction analyses were conducted. However, exploratory comparisons of gut microbiota composition were performed across BMI categories and by early-life exposures. According to the body condition data obtained (weight/height ratio), a high percentage of normal-weight children were found (73 %) with no significant differences between sexes (p=0.709) lines 200-203. |
| Discussion | | |  |
| Key results | 18 | Summarise key results with reference to study objectives | Lines 252-262, 270-271, 277-279, 289-292 |
| Limitations | 19 | Discuss limitations of the study, taking into account sources of potential bias or imprecision. Discuss both direction and magnitude of any potential bias | Lines 340-345 |
| Interpretation | 20 | Give a cautious overall interpretation of results considering objectives, limitations, multiplicity of analyses, results from similar studies, and other relevant evidence | Lines 352-362 |
| Generalisability | 21 | Discuss the generalisability (external validity) of the study results | Lines- 346-348 |
| Other information | | |  |
| Funding | 22 | Give the source of funding and the role of the funders for the present study and, if applicable, for the original study on which the present article is based | This work was supported by Biocodex Microbiota Foundation, through the Henri Boulard Award, and Consejo Veracruzano de Investigación Científica y Desarrollo Tecnológico (COVEICYDET) for the resources allocated to the development of this project. |

*Give information separately for exposed and unexposed groups.

**Note:** An Explanation and Elaboration article discusses each checklist item and gives methodological background and published examples of transparent reporting. The STROBE checklist is best used in conjunction with this article (freely available on the Web sites of PLoS Medicine at http://www.plosmedicine.org/, Annals of Internal Medicine at http://www.annals.org/, and Epidemiology at http://www.epidem.com/). Information on the STROBE Initiative is available at www.strobe-statement.org.
